# The antecedents of women’s external locus of control: Associations with characteristics of their parents and their early childhood

**DOI:** 10.1016/j.heliyon.2017.e00236

**Published:** 2017-01-30

**Authors:** Jean Golding, Steven Gregory, Yasmin Iles-Caven, Stephen Nowicki

**Affiliations:** a Centre for Child and Adolescent Health, School of Social and Community Medicine, University of Bristol, Bristol, UK; b Department of Psychology, Emory University, Atlanta, GA, USA

**Keywords:** Psychology

## Abstract

Locus of control (LOC) measures individuals’ expectancies regarding their ability to affect what happens to them based on how they behave. The more they believe their behaviour has something to do with what happens to them the more internal they are. In contrast the more they perceive that what happens to them is beyond their control and determined by luck, fate, chance or powerful others the more external they are. Copious research findings suggest that external LOC (ELOC) is associated with many adverse personal, social, academic and health outcomes. In spite of its importance in so many areas of human behaviour relatively little is known about the features of the early background of individuals that contributes to these expectancies. This is the first in a number of studies that will suggest possible antecedents and consequences of having a high ELOC.

The study takes advantage of the data collected in the Avon Longitudinal Study of Parents and Children (ALSPAC), which started by studying pregnancies in 1991–1992 of residents in an area of south-west England. Over 12000 of the women who enrolled during pregnancy completed a set of questions in mid-pregnancy from which an LOC score was computed. ELOC was defined as a score greater than the median. The relationships with characteristics of the women’s parents and her early childhood (<6 years) are considered first as unadjusted odds ratios and then as adjusted after analysis using hierarchical sets of stepwise logistic regressions. The relative contributions to the women’s ELOC was measured using a goodness-of-fit (GOF) measure.

The analyses demonstrated the independent importance of maternal and paternal backgrounds as well as features of her early childhood (<6 years). The final model identified nine independent features (each with *P* < 0.0001): year of birth of her mother, maternal and paternal education levels, father smoked, mother smoked when pregnant, year of birth of study woman, the number of older siblings she had, whether her father was absent during this period, and whether she spent her childhood in the study area.

In conclusion, the woman’s LOC appears to be independently influenced by a number of characteristics which may give clues as to possible mechanisms—and how internality may be supported in the future. Subsequent papers will assess both whether features of later childhood influence the woman’s LOC and whether the LOC of men in the study have similar antecedents.

## 1 Introduction

Locus of control (LOC) refers to individuals’ generalized expectancy regarding the connection between their behaviour and its consequences in a problem solving context. Those who fail to see a connection between what they do and what happens to them and instead view what happens to them as the result of luck, fate, chance, or powerful others are seen as externally controlled (ELOC). Conversely those who tend to perceive a connection between their efforts and what happens are called internally controlled (ILOC).

Because of 800+ definitions of “locus of control” that are sprinkled throughout the literature, it is important that each study clearly state the definition of the locus of control being used (Skinner, 1996). Peterson and Stunkard (1992) noted the possible confusion that could result from using efficacy and perceived control (Bandura, 1986; Infurna and Mayer, 2015; Lachman and Weaver, 1998) or attribution (Peterson and Seligman, 1984; Seligman, 1975) as though they were synonymous with locus of control of reinforcement as defined by Rotter who saw it as a generalized expectancy within his social learning theory (1954, 1966). As Peterson and Stunkard (1992) put it:

“Locus of control refers to one’s generalized expectancies about the origin of rewards and punishment in the world; self-efficacy refers to one’s belief about whether a given behavior can be enacted and explanatory style refers to one’s habitual way of explaining the causes of events.” (p. 115).

Although each cognate has generated a significant and extensive set of findings in its own right, it is important to remember that because each comes from a different theoretical perspective it may be measuring something somewhat different from the others. In the present study we are defining locus of control of reinforcement as the cognate introduced by Rotter (1966).

Over the past 50 years since its introduction, LOC as defined by Rotter has proven to be one of the most popular variables for researchers who have found it to be significantly related to an ever growing number of important and significant aspects of human life including personality characteristics, social adjustment (Chipperfield et al., 2016), academic achievement (Shepherd et al., 2006), health (Zampieri and de Souza, 2011) and business success (Kormanik and Rocco, 2009). For additional reviews of associations with locus of control see Lefcourt, (1981, 1983), Nowicki, (2016a), Nowicki and Duke (2016), Rotter (1966) and Rotter (1975; 1990).

Rotter (1966) and Rotter (1975; 1990) offered clear theoretical assumptions for the development of LOC expectancies. For him the basic LOC orientations are initially learned through children’s experiences with their parents. To facilitate the learning of internal LOC Rotter suggested parents to (1) consistently reinforce children’s behaviour contingently, (2) allow children more autonomy and independence and (3) create a nurturing safe environment within which children can discover the connections between how they behave and the consequences. Carton and Nowicki reviewed the extant literature in 1994 to evaluate whether these theorized antecedents of LOC were supported. They concluded that there was empirical support for four parental factors in the development of children’s LOC: (a) The degree of control parents exhibited over their children: more control, higher externality, less control more internality. (b) Externality was associated with a greater degree of life stress produced by father absence due to divorce or death and/or by intense marital discord. (c) Children’s internality was associated with parents who were perceived by children or by themselves as warm, emotionally supportive and nurturing. (d) Internality was associated with parents who rewarded and punished consistently and contingently. However, Carton and Nowicki (1994) noted that these conclusions were based on data gathered from research studies that used relatively few participants from homogeneous populations of participants.

One exception comes from a more recent study. Wickline et al. (2011) found support for many of these associations, especially regarding the role of non-authoritarian parenting style in the analysis of data from a British longitudinal cohort study of mothers and their children. In this paper, which will be the first of a suite of papers on the factors influencing LOC orientation and its consequences, we investigate the very early antecedent factors that relate to the development of externally controlled women at the time in which they are anticipating the birth of their study child. We see this as a prelude to an analysis of factors associated with the development of features of LOC in their partners and their children. In particular we assess the extent to which their early background, including events in their own childhood, and characteristics of their parents, are associated with each woman’s LOC orientation.

## 2 Material and methods

### 2.1 The ALSPAC study

This pre-birth cohort was designed to determine the environmental and genetic factors that are associated with health and development of the study offspring (Fraser et al., 2013; Golding and ALSPAC Study Team, 2004). As part of the study design, therefore, there was a concerted effort before the child’s birth to obtain from the parents details of their personalities, moods and attitudes, including a measure of their LOC.

ALSPAC recruited 14,541 pregnant women resident in Avon, UK with expected dates of delivery between 1st April 1991 and 31st December 1992 (an estimated 80% of the eligible population). Data were collected at various time-points using self-completion questionnaires, biological samples, hands-on measurements, and linkage to other data sets. For full details of all the data collected see the study website: www.bristol.ac.uk/alspac/researchers/data-access/data-dictionary/. Ethical approval for the study was obtained from the ALSPAC Ethics and Law Committee and the Local Research Ethics Committees.

For this project we concentrate on the data collected from questionnaires completed before the birth of the study child. The pregnant women were sent four questionnaires during the pregnancy, one of which contained the LOC scale.

### 2.2 The outcome measure

The locus of control measure used in the present study is a shortened version of the adult version of the Nowicki-Strickland Internal-External locus of control scale (ANSIE). The ANSIE (Nowicki and Duke, 1974) comprises 40 items in a yes/no format, which assess perceived control. This measure was chosen over other scales more specifically related to perceived control over health, as it was considered that this more generalized scale would relate to other factors in addition to health outcomes. Construct validity for the scale has been found in the results of over a thousand studies (Nowicki, 2016b). The version used in the present study comprises 12 of the original 40 items which were chosen after factor analysis of the ANSIE administered as a pilot to 135 mothers. The 12 questions loaded onto a single factor of general locus of control. The 12 questions used are shown in Table 1. From the responses from 12,471 women a ‘locus of control score’ was derived, the higher the score the more external the locus of control. The scores ranged from 0 to 12. The frequency was normally distributed with a median of 4 (Table 2). For this study external locus of control was defined as having a score of >4. This cut-off identified 45.2% of the women as externally controlled (ELOC).

**Table 1:** The twelve yes/no questions making up the ALSPAC Locus of Control score.

1.	Did getting good marks at school mean a great deal to you?
2.	Are you often blamed for things that just aren’t your fault?
3.	Do you feel that most of the time it doesn’t pay to try hard because things never turn out right anyway?
4.	Do you feel that if things start out well in the morning that it’s going to be a good day no matter what you do?
5.	Do you believe that whether or not people like you depends on how you act?
6.	Do you believe that when bad things are going to happen they are just going to happen no matter what you try to do to stop them?
7.	Do you feel that when good things happen they happen because of hard work?
8.	Do you feel that when someone doesn’t like you there’s little you can do about it?
9.	Did you usually feel that it was almost useless to try in school because most other children were cleverer than you?
10.	Are you the kind of person who believes that planning ahead makes things turn out better?
11.	Most of the time, do you feel that you have little to say about what your family decides to do?
12.	Do you think it’s better to be clever than to be lucky?

[N.B. For creating the LOC score, questions 2, 3, 4, 6, 8, 9 and 11 were coded as yes = 0, no = 1; the remaining questions were coded as yes = 1, no = 0. The responses were then summed].

**Table 2:** Distribution of the locus of control score of the pregnant women.

LOC Score	N (%)	Cumulative %
0	229 (1.8)	1.8
1	759 (6.1)	7.9
2	1506 (12.1)	20.0
3	1988 (15.9)	35.9
4	2353 (18.9)	54.8
5	2064 (16.6)	71.4
6	1594 (12.8)	84.1
7	1011 (8.1)	92.2
8	547 (4.4)	96.6
9	263 (2.1)	98.7
10	120 (1.0)	99.7
11	33 (0.3)	100.0
12	4 (< 0.1)	100.0

**Total**	**12471 (100.0)**	**100.0**

### 2.3 The variables considered

In this paper we consider three different groups of variables pertaining to: (a) the demographic background of the mothers of each woman; (b) the demographic background of their fathers; and (c) their birth and early childhood (<6 years). The definitions of most variables used are standard, and data relevant to the parents of the women are outlined below.

#### 2.3.1 Education

Information was obtained on all the qualifications of the woman’s mother and her father. From the information obtained a 5-point education scale has been obtained for each, with the following categories: No qualifications; Not higher than CSE or GCSE (D,E,F or G); O-Level or equivalent; A-level or equivalent, such as Teaching or Nursing qualification; University degree. This scale was similar to that derived for the Child Health & Education Study (Osborn et al., 1984). For the present study, these qualifications have been categorised into two groups: O-level and above; lower than O-level.

#### 2.3.2 Occupation

Data were obtained concerning the employment situation of her mother and her father with details of the normal job, occupation, trade or profession with the type of industry or service given. These occupations were classified using the Standard Occupational Classification (SOC) codes published by the Employment Department Group Office of Population Censuses and Surveys of Great Britain (Office of Population Censuses and Surveys, 1990). The SOC divides occupations into groups based upon the qualifications and skills necessary to perform each job optimally.

#### 2.3.3 Ethnic origins

The ethnic origins of the woman and her parents were obtained using the format asked in the 1991 United Kingdom Census. This categorises the person as White, Black/Caribbean, Black/African, Black/Other, Indian, Pakistani, Bangladeshi, Chinese, Other Specified. In the Avon area at this time, about 6% of the population comprised ethnic minorities.

#### 2.3.4 Childhood happiness

Developed by the ALSPAC team the woman was asked: ‘Looking back would you call your childhood happy?’ for three age groups, with the options ‘yes very happy’, ‘yes moderately happy’, ‘not really happy’, ‘no quite unhappy’, ‘no very unhappy’.

### 2.4 Statistical analyses

**Fig. 1:**
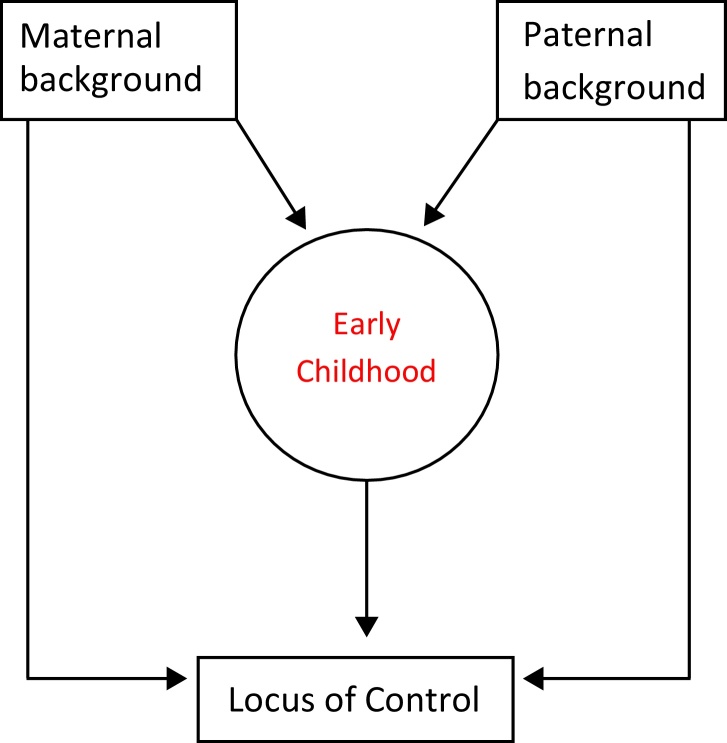
Theoretical depiction of the way in which the parental backgrounds and early childhood might influence the woman’s locus of control.

The research aims were:

(i) to assess the extent to which different aspects of the backgrounds of the parents are associated with the ELOC of the woman;
(ii) to determine whether features of the first 5 years of her life are related to the woman’s ELOC;
(iii) to assess whether the demographic features of the parents influence (act through) features of the early childhood to impact the woman’s risk of having an ELOC (see Fig. 1).

The following analyses were undertaken: (i) the unadjusted associations with ELOC were calculated for each of the three groups of variables; (ii) the variables with unadjusted *P* value < 0.05 were selected and offered to a backward logistic regression for each group; (iii) the results for each group were considered in regard to the numbers of individuals left in each regression and variables were either dropped or recoded to increase the numbers available in the regression where feasible; (iv) once these intra-domain regressions were finalized, the groups were combined for inter-group analyses in a similar way to our earlier publications (e.g. Golding et al., 2014). Comparison of goodness-of-fit (GOF) between the analyses used 100 times the pseudo-R^2^ statistic, the higher the value the better the fit.

## 3 Results

### 3.1 Characteristics of the background of their mothers

#### 3.1.1 Unadjusted analyses

The following variables were considered in relation to the mothers: the proportion of women who had an ELOC in regard to their year of birth; ethnic group; educational level achieved; whether they had ever smoked and (for their mothers only) if so whether they had smoked when pregnant with the study woman; their ages at birth of the study woman; and their social group based on occupation. The unadjusted associations with the proportion of women with ELOC are depicted in Table 3. There were significant unadjusted associations of ELOC with her mother’s year of birth, such that the more recently her mother had been born, especially if during or after the Second World War, the greater the risk; the risk was also increased if her mother had a low education level, young age at giving birth to her daughter, a history of smoking, including whether she had smoked when pregnant with her daughter, and lower social group based on a classification of her occupation. There was no association with ethnic group of her mother and this variable has not been considered further.

**Table 3:** Proportion (no.) of women externally controlled tabulated against demographic features of their parents.

Features of their parents	MOTHERS			FATHERS		
	%(n) ELOC	OR [95% CI]	*P*	%(n) ELOC	OR [95% CI]	*P*
*Year of Birth*	N = 10647		<0.0001	N = 9876		<0.0001

Pre 1925	31.6% (302)	0.77 [0.65, 0.91]		33.1% (555)	0.84 [0.73, 0.96]	
1925–1929	34.9% (465)	0.89 [0.77, 1.03]		36.1% (587)	0.96 [0.84, 1.10]	
1930–1934	37.5% (768)	1.00 Ref		37.0% (772)	1.00 Ref	
1935–1939	38.0% (896)	1.02 [0.91, 1.16]		41.5% (853)	1.21 [1.06, 1.37]	
1940–1944	46.3% (967)	1.44 [1.27, 1.63]		48.0% (696)	1.57 [1.37, 1.80]	
Post 1944	59.0% (1103)	2.40 [2.11, 2.73]		61.7% (611)	2.73 [2.34, 3.19]	

*Ethnic Group*	N = 11728		0.937	N = 11686		0.434
White	44.1% (5065)	1.00 Ref		44.1% (5029)	1.00 Ref	
Non-white	43.9% (107)	0.99 [0.77, 1.28]		41.7% (116)	0.91 [0.71, 1.16]	

*Education Level*	N = 8873		<0.0001	N = 8424		<0.0001
≥O-Level	31.1% (962)	1.00 Ref		30.9% (977)	1.00 Ref	
<O-Level	47.0% (2712)	1.96 [1.78, 2.17]		47.7% (2511)	2.04 [1.85, 2.22]	

*Age at birth of woman*	N = 10656		<0.0001	N = 9889		<0.0001
<25	48.2% (2027)	1.51 [1.39, 1.64]		50.1% (1068)	1.59 [1.44, 1.76]	
25-34	38.0% (1981)	1.00 Ref		38.7% (2147)	1.00 Ref	
35+	40.0% (497)	1.09 [0.96, 1.23]		39.4% (511)	1.03 [0.91, 1.17]	

*Ever Smoked*	N = 11816		<0.0001	N = 11626		<0.0001
Yes	49.0% (3252)	1.47 [1.37, 1.58]		47.0% (4159)	1.50 [1.37, 1.64]	
No	39.5% (2045)	1.00 Ref		37.1% (1028)	1.00 Ref	

*Smoked when pregnant with subject*	N = 11769		<0.0001		N/A	
Yes	52.5% (2293)	1.64 [1.52, 1.77]				
No	40.2% (2978)	1.00 Ref				

*Social group*	N = 6573		<0.0001	N = 9634		<0.0001
Higher managerial	36.6% (48)	1.08 [0.74, 1.59]		27.4% (399)	0.69 [0.60, 0.80]	
Lower managerial	34.8% (306)	1.00 Ref		35.2% (847)	1.00 Ref	
Intermediate	42.0% (519)	1.35 [1.13, 1.62]		37.7% (204)	1.11 [0.92, 1.35]	
Small employers	38.3% (80)	1.16 [0.85, 1.59]		48.1% (515)	1.70 [1.47, 1.97]	
Lower supervisory	39.6% (19)	1.23 [0.68, 2.22]		46.2% (1101)	1.58 [1.41, 1.78]	
Semi-routine	52.3% (678)	2.05 [1.72, 2.45]		48.0% (333)	1.70 [1.43, 2.01]	
Routine	58.2% (506)	2.61 [2.15, 3.17]		56.1% (607)	2.35 [2.03, 2.72]	

#### 3.1.2 Adjusted analyses

Because of missing data in the social grouping, an additional category was added to the social group to indicate ‘housewives’; for the low education group, those with missing data were included in the low category. On mutual adjustment (Table 4) two variables dropped out of the model: the variable concerning ever smoked dropped out in favour of the variable concerning whether the mother had smoked when pregnant; and the mother’s social group ceased to be significant in the presence of her education level. All other variables were retained in the model, but mutual adjustment had resulted in the relationship with maternal youth being reversed; thus on adjustment, the daughters of women who had been <25 years old when they gave birth had a decreased risk once their mothers’ year of birth had been taken into account.

**Table 4:** Backwards step-wise logistic regression of the woman’s locus of control score (>4 versus ≤4) using the variables relating to her mother’s background.

Characteristics of her mother	Univariable			Intra domain adjustment		
	N	*P*	OR [95% CI]	N	*P*	OR [95% CI]
Mother’s year of birth	10642	<0.0001****	1.43 [1.37, 1.48]	10642	<0.0001****	1.50 [1.42, 1.58]
Mother’s education < O-Level	11949	<0.0001****	2.13 [1.96, 2.33]	10642	<0.0001****	2.00 [1.82, 2.17]
Mother smoked	11811	<0.0001****	1.47 [1.37, 1.58]	10168	0.153	1.08 [0.97, 1.21]
Mother aged <25 at birth of woman	10651	<0.0001****	1.49 [1.38, 1.61]	10642	<0.001***	0.83 [0.74, 0.93]
Mother’s social group	12633	0.017*	0.63 [1.00, 1.03]	10642	0.826	1.00 [0.98, 1.01]
Smoked when pregnant with woman	12633	<0.0001****	1.63 [1.52, 1.76]	10642	<0.0001****	1.37 [1.26, 1.48]

Total N = 10642; Overall GOF = 4.34%.

### 3.2 Characteristics of the background of their fathers

#### 3.2.1 Unadjusted analyses

Characteristics of the woman’s father were related to her ELOC in a similar way to that of her mother: the more recently he had been born, if he had a low level of education, a history of smoking, was aged <25 at the birth of his daughter or was in a lower social group based on his occupation the more likely was his daughter to be externally oriented (Table 3).

#### 3.2.2 Adjusted analyses

As with the mothers, there were data missing in regard to education and social group. The education variable was treated in the same way as for the study women. Stepwise logistic regression including this group of variables resulted in all being included, but again the reversal of risk occurred on adjustment for the fathers aged <25 at the birth of the study woman (Table 5).

**Table 5:** Backwards step-wise logistic regression of the woman’s locus of control score (>4 versus ≤4) using the variables relating to her father’s background.

Father’s background	Univariable			Intra domain		
	N	*P*	OR [95% CI]	N	*P*	OR [95% CI]
Father’s year of birth	9876	<0.0001****	1.38 [1.32, 1.43]	8110	<0.0001****	1.42 [1.33, 1.50]
Father’s education < O-Level	12633	<0.0001****	2.22 [2.04, 2.44]	8110	<0.0001****	1.43 [1.27, 1.61]
Father smoked	11621	<0.0001****	1.50 [1.37, 1.64]	8110	<0.0001****	1.31 [1.18, 1.47]
Father aged <25 at birth of woman	9885	<0.0001****	1.58 [1.44, 1.74]	8110	0.004**	0.80 [0.69, 0.93]
Father’s social group	9632	<0.0001****	1.20 [1.17, 1.22]	8110	<0.0001****	1.13 [1.10, 1.16]

Total N = 8110, Overall GOF = 4.64.

### 3.3 Both parents considered together

When both the maternal and paternal features were considered together, only one dropped from the analysis—paternal age <25 (Table 6); all other variables were retained in the analysis indicating that they were independent contributors. Thus, year of birth of each parent was important, although the effect size was greater for the mothers; education level was important for both parents, with similar effect sizes, those who were more educated having a daughter less likely to be externally oriented; smoking was independently associated with ELOC, with prenatal smoking by the mother and paternal smoking history having similar associations.

**Table 6:** Backwards step-wise logistic regression of the woman’s locus of control score (>4 versus ≤4) using the variables relating to background of both her mother and father.

Variable	Univariable			Intra domain		
	N	*P*	OR [95% CI]	N	*P*	OR [95% CI]
Mother’s year of birth	10642	<0.0001****	1.43 [1.37, 1.48]	8062	<0.0001****	1.32 [1.21, 1.45]
Mother’s education < O-Level	11949	<0.0001****	2.13[1.96, 2.33]	8062	<0.0001****	1.43 [1.27, 1.61]
Mother aged <25 at birth of woman	10651	<0.0001****	1.49 [1.38, 1.61]	8062	<0.0001****	0.77 [0.67, 0.87]
Mother smoked when pregnant	12633	<0.0001****	1.63 [1.52, 1.76]	8062	<0.0001****	1.25 [1.13, 1.38]
Year of birth of father	9876	<0.0001****	1.38 [1.32, 1.43]	8062	<0.001***	1.15 [1.06, 1.24]
Father’s education < O-Level	12633	<0.0001****	2.22 [2.04, 2.44]	8062	<0.0001****	1.33 [1.16, 1.52]
Father was a smoker	11621	<0.0001****	1.50 [1.37, 1.64]	8062	<0.0001****	1.26 [1.13, 1.41]
Father aged <25 at birth of woman	9885	<0.0001****	1.58 [1.44, 1.74]	8062	0.102	0.87 [0.74, 1.03]
Social group of father	9632	<0.0001****	1.20 [1.17, 1.22]	8062	<0.0001****	1.12 [1.09, 1.15]

Total N = 8062, Overall GOF = 5.43.

### 3.4 Relationship with facets of her early childhood (≤5 years)

Information collected includes estimates of the woman’s ethnic background, area of residence at the time she was born, whether she had a birthmark, was adopted in the first year of life, whether breastfed, and the number of older siblings she had. Additional information collected for the first 5 years of life included whether any of the following had been present in the home: mother, father, step-father, step-brother, step-sister, mother’s partner or father’s partner. Other details concerning the first 5 years included whether parents had divorced or separated, or whether a parent had died. Finally she had been asked to rate her degree of happiness during this part of her childhood, using a 5-part scale.

#### 3.4.1 Unadjusted analyses

Univariable analysis identified 14 of the 18 variables to be statistically significant (Table 7); only her ethnic background, being adopted in the first year, whether her father had died, and whether her mother’s partner was present were omitted from further analysis. The statistically significant variables included increased risk of ELOC if she had a birthmark, her year of birth − the more recently she was born the higher the risk, the number of older siblings she had, whether her mother had died during this time, divorce or separation of her parents, mother absent or father absent from the home, presence of step-father or step-sibling, and rating of unhappiness were all positively related to ELOC, and residence outside Avon and being breast fed were negatively associated.

**Table 7:** Proportion of women externally controlled tabulated against features of their early childhood (≤5yrs).

Features of early childhood			
	%(n) ELOC	OR [95% CI]	*P*
***In First Year***			
*Year of her birth*	N = 12564		<0.0001
<1955	33.9% (169)	0.77 [0.63, 0.93]	
1955–1959	34.6% (750)	0.79 [0.71, 0.88]	
1960–1964	40.1% (1917)	1.00 Ref	
1965–1969	51.7% (1972)	1.60 [1.47, 1.74]	
1970+	67.1% (872)	3.06 [2.68, 3.48]	

*Had a birthmark*	N = 12448		<0.0001
Yes	49.1% (1541)	1.23 [1.13, 1.33]	
No	43.9% (4088)	1.00 Ref	

*Ethnic background*	N = 11817		0.686
White	44.2% (5096)	1.00 Ref	
Non-white	45.4% (128)	1.05 [0.83, 1.33]	

*Place of residence*	N = 11534		<0.0001
Avon	52.7% (3403)	1.00 Ref	
Rest of England	35.4% (1382)	0.49 [0.45, 0.53]	
Rest of World	34.0% (396)	0.46 [0.39, 0.55]	

*Was adopted in first year*	N = 12638		0.346
Yes	49.0% (75)	1.17 [0.85, 1.60]	
No	45.2% (5643)	1.00 Ref	

*Was breastfed*	N = 10007		<0.0001
Yes	39.2% (2266)	0.73 [0.67, 0.79]	
No	47.0% (1987)	1.00 Ref	

*No. of Older Siblings*	N = 11937		<0.0001
0	42.1% (1580)	1.00 Ref	
1	44.0% (2116)	1.08 [0.99, 1.18]	
2	44.6% [989)	1.11 [0.99, 1.23]	
3	51.7% (379)	1.47 [1.25, 1.72]	
4+	56.5% (236)	1.78 [1.45, 2.19]	
***In First 5 Years***			

*Mother died*	N = 12637		<0.0001
Yes	71.7% (43)	3.08 [1.75, 5.40]	
No	45.1% (5674)	1.00 Ref	

*Father died*	N = 12636		0.474
Yes	41.7% (43)	0.87 [0.58, 1.28]	
No	45.3% (5674)	1.00 Ref	

*Mother Present in Home*	N = 12638		<0.0001
Yes	44.1% (5258)	0.44 [0.38, 0.52]	
No	64.0% (460)	1.00 Ref	

*Father Present in Home*	N = 12638		<0.0001
Yes	43.6% (5048)	0.44 [0.38, 0.50]	
No	63.9% (670)	1.00 Ref	

*Step-father Present in Home*	N = 12638		0.006
Yes	56.7% (80)	1.60 [1.14, 2.23]	
No	45.1% (5638)	1.00 Ref	

*Step-brother Present in Home*	N = 12638		0.005
Yes	60.2% (53)	1.84 [1.20, 2.82]	
No	45.1% (5665)	1.00 Ref	

*Step-sister Present in Home*	N = 12638		0.011
Yes	59.7% (46)	1.80 [1.14, 2.85]	
No	45.2% (5672)	1.00 Ref	

*Mother’s Partner Present*	N = 12638		0.016
Yes	61.4% (35)	1.93 [1.13, 3.30]	
No	45.2% (5683)	1.00 Ref	

*Father’s Partner Present*	N = 12638		0.077
Yes	61.3% (19)	1.92 [0.93, 3.96]	
No	45.2% (5699)	1.00 Ref	

*Parents Divorced/Separated*	N = 12424		<0.0001
Yes	62.4% (402)	2.09 [1.77, 2.46]	
No	44.3% (5220)	1.00 Ref	

*Recollection of happiness*	N = 11629		<0.0001
Very happy	42.1% (3945)	1.00 Ref	
Moderately happy	52.5% (1006)	1.52 [1.38, 1.68]	
Not really happy	60.0% (138)	2.06 [1.58, 2.69]	
Quite unhappy or very unhappy	63.2% (72)	2.36 [1.61, 3.46]	

#### 3.4.2 Adjusted analyses

Backwards stepwise analysis revealed just six of the 14 variables to be independently associated (Table 8): having a birthmark, year of birth, residence in Avon, having been breast fed, number of older siblings, and father being absent from the home. Mother being absent from the home was of borderline significance only (*P* = 0.077). The overall GOF statistic was 5.8 (n = 8614).

**Table 8:** Backwards step-wise logistic regression of the woman’s locus of control score (>4 versus ≤4): her early childhood.

Features of early childhood	Univariable			Intra domain		
	N	P	OR [95% CI]	N	P	OR [95% CI]
Has birthmark	12638	<0.0001****	1.23 [1.13, 1.33]	8614	0.002**	1.18 [1.06, 1.30]
Year of birth of woman	12564	<0.0001****	1.79 [1.70, 1.89]	8614	<0.0001****	1.65 [1.54, 1.77]
Lived in Avon	11534	<0.0001****	2.06 [1.91, 2.22]	8614	<0.0001****	1.80 [1.64, 1.97]
Was breast fed	10007	<0.0001****	0.73 [0.67, 0.79]	8614	<0.001***	0.85 [0.78, 0.93]
Number of older siblings	11937	<0.0001****	1.33 [1.22, 1.44]	8614	<0.0001****	1.27 [1.13, 1.41]
Mother present in household	12638	<0.0001****	0.44 [0.38, 0.52]	8614	0.072	0.74 [0.54, 1.03]
Father present in household	12638	<0.0001****	0.43 [0.38, 0.50]	8614	<0.0001****	0.53 [0.44, 0.64]
Stepfather present in household	12638	0.006**	1.60 [1.14, 2.23]	8614	0.156	0.72 [0.45, 1.14]
Step-siblings present in household	12638	0.004**	1.69 [1.18, 2.43]	8614	0.564	1.17 [0.68, 2.02]
Mother died	12637	<0.0001****	3.08 [1.75, 5.40]	8614	0.218	1.84 [0.70, 4.85]
Parents divorced/separated	12424	<0.0001****	2.09 [1.77, 2.46]	8506	0.670	1.05 [0.83, 1.34]
Unhappiness in early childhood	12637	<0.0001****	3.08 [1.75, 5.40]	8614	0.218	1.84 [0.70, 4.85]

Total N = 8614, GOF = 5.79.

### 3.5 Parental and early childhood variables combined

In Table 9 we provide the results of combining the four significant variables from Table 4 with the six from Table 8 to determine whether the childhood characteristics explained any of the maternal ones. This shows that although the years of birth of the woman and her mother are retained, that of maternal age <25 ceases to be significant. In the presence of maternal prenatal smoking the breast fed variable ceases to be associated (the relationship between prenatal smoking and failure to breast feed is well documented (Scott and Binns, 1998)), and the presence of a birthmark becomes only marginally significant. The highest odds ratios concern the absence of her father during this part of childhood (1.87), and poor maternal education level (1.72), followed by residence in Avon (1.58) and her year of birth (1.40).

**Table 9:** Backwards logistic regression combining maternal characteristics with those of the early childhood of the woman.

Characteristics of mother and details of early childhood	Univariable			Intra domain		
	N	*P*	OR [95% CI]	N	*P*	OR [95% CI]
Year of birth of her mother	10642	<0.0001****	1.43 [1.37, 1.48]	9855	<0.0001****	1.18 [1.12, 1.24]
Education < O-Level of her mother	11949	<0.0001****	2.13 [1.96, 2.33]	9855	<0.0001****	1.72 [1.56, 1.92]
Mother aged <25 at birth of woman	10651	<0.0001****	1.49 [1.38, 1.61]	9855	0.860	1.01 [0.89, 1.15]
Mother smoked when pregnant	12633	<0.0001****	1.63 [1.52, 1.76]	9855	<0.0001****	1.25 [1.15, 1.37]
Has birthmark	12633	<0.0001****	1.23 [1.13, 1.33]	9855	0.049*	1.10 [1.00, 1.21]
Year of birth of index woman	12559	<0.0001****	1.79 [1.70, 1.89]	9855	<0.0001****	1.40 [1.29, 1.51]
Lived in Avon	11531	<0.0001****	2.06 [1.91, 2.22]	9855	<0.0001****	1.58 [1.45, 1.72]
Breast fed	10003	<0.0001****	0.73 [0.67, 0.79]	8485	0.053	0.91 [0.83, 1.00]
Number of older siblings	11932	<0.0001****	1.33 [1.22, 1.45]	9855	<0.0001****	1.31 [1.19, 1.46]
Father absent from household	12633	<0.0001****	2.30 [2.01, 2.62]	9855	<0.0001****	1.87 [1.58, 2.23]

Total N = 9855, Overall GOF = 6.40

Total N = 6615, Overall GOF = 6.43.

A similar approach was taken to determine the way in which paternal variables in Table 5 might influence those in Table 8 in regard to the risk of the woman having an ELOC. The results indicated that the paternal age <25 variable was an indicator of the year at which the woman was born and ceased to enter. Having a birthmark also ceased to enter (Table 10). The GOF for this model was 6.43 for a relatively smaller sample of 6615.

**Table 10:** Backwards logistic regression combining paternal characteristics with those of the early childhood of the woman.

Characteristics of father and details of early childhood	Univariable			Intra domain		
	N	*P*	OR [95% CI]	N	*P*	OR [95% CI]
Year of birth of father	9876	<0.0001****	1.38 [1.32, 1.43]	6615	0.004**	1.10 [1.03, 1.18]
Father’s education <O-Level	12633	<0.0001****	2.22 [2.04, 2.44]	6615	<0.0001****	1.39 [1.23, 1.59]
Father smoked	11621	<0.0001****	1.50 [1.37, 1.64]	6615	<0.001***	1.26 [1.12, 1.43]
Father aged <25 at birth of woman	9885	<0.0001****	1.58 [1.44, 1.74]	6615	0.933	0.99 [0.83, 1.18]
Father’s social group	9632	<0.0001****	1.20 [1.17, 1.22]	6615	<0.0001****	1.11 [1.07, 1.14]
Has birthmark	12633	<0.0001****	1.23 [1.13, 1.33]	6615	0.103	1.11 [0.98, 1.25]
Year of birth of woman	12559	<0.0001****	1.79 [1.70, 1.89]	6615	<0.0001****	1.53 [1.37, 1.70]
Lived in Avon	11531	<0.0001****	2.06 [1.91, 2.22]	6615	<0.0001****	1.52 [1.36, 1.70]
Breast fed	10003	<0.0001****	0.73 [0.67, 0.79]	6615	0.017*	0.88 [0.79, 0.98]
Number of older siblings	11932	<0.0001****	1.33 [1.22, 1.45]	6615	0.011*	1.20 [1.04, 1.37]
Father absent from household	12633	<0.0001****	2.30 [2.01, 2.62]	6615	0.036*	1.35 [1.02, 1.79]

### 3.6 The final model

In order to maximise the numbers of individuals in the final model, we omitted the variables with high numbers of missing data and which were likely to skew the results; for example the study woman was unlikely to be able to record the information relating to her father’s social group if he had been absent from the family home, nor would standard missing data techniques be able to cope with this problem. We have therefore omitted those variables that would be likely to both reduce the numbers available and the validity of the results concerning presence of the father, and retained just two—paternal education and paternal smoking, where the unknown responses have been coded to ‘no’. Thus the paternal education variable should be interpreted as exposed to an educated father, and paternal smoking becomes exposed to paternal smoking.

The final model has retained nine variables: three concerning her mother, two concerning her father, and four relating to her early childhood. Dropped from the analysis were the variables maternal age and having a birthmark (Table 11).

**Table 11:** FINAL MODEL: Backwards logstic regression combining maternal and paternal characteristics with those of the early childhood of the woman.

Parental and early child characteristics	Intra domain		
	N	*P*	OR [95% CI]
Year of birth of mother	9286	<0.0001****	1.20 [1.13, 1.26]
Maternal education < O-Level	9286	<0.0001****	1.43 [1.27, 1.61]
Mother smoked when pregnant	9286	<0.0001****	1.23 [1.12, 1.35]
Paternal education < O-Level	9286	<0.0001****	1.37 [1.22, 1.54]
Father smoked	9286	<0.0001****	1.27 [1.14, 1.41]
Year of birth of study woman	9286	<0.0001****	1.40 [1.29, 1.52]
Lived in Avon	9286	<0.0001****	1.56 [1.43, 1.71]
Number of older siblings	9286	<0.0001****	1.28 [1.15, 1.42]
Father absent from household	9286	<0.0001****	1.81 [1.49, 2.19]

Total N = 9286, Overall GOF = 6.89.

### 3.7 Goodness of fit

The way in which the GOF statistics varies with each group of characteristics in each model demonstrates that each of the three groups has an impact by increasing the GOF in combination with the other group(s) (Table 12). This implies that all (i.e. characteristics of mothers, fathers and early childhood) have an independent association with the woman’s risk of having an ELOC.

**Table 12:** Comparisons of goodness of fit (GOF) for different adjusted models.

Model	No. in model	GOF
Maternal characteristics (MC)	10,642	4.34
Paternal characteristics (PC)	8,110	4.64
Early childhood (EC)	8,614	5.79
MC + PC	8,062	5.43
MC + EC	9,855	6.40
PC + EC	6,615	6.43
MC + PC + EC	9,286	6.89

## 4 Discussion

In this paper we have taken a hypothesis-free approach to determining ways in which the details of the woman’s parents and her early childhood are associated with her ELOC score similar to approaches we have used with other outcomes in ALSPAC (e.g. Golding et al., 2014). Our research questions concerned firstly to assess the ways in which the features of her parents were associated with her ELOC; secondly to determine features of her early childhood which predicted her risk of ELOC; and thirdly to assess whether features of the parents explained the associations with early childhood (and vice versa). We have shown that the demographic background of each parent was independently associated with ELOC, and that very few of the early childhood variables were ‘explained’ by the parents’ backgrounds. We discuss the results for each set of variables below.

### 4.1 Year of birth and ages of parents

Although it is normal to consider the parent’s age as an indicator of maturity, as well as of increased social capital, the year in which the individual was born can also provide a different indication of their political, environmental and socio-economic backgrounds. In this study we found that the later the birth of a parent the higher the risk of the daughter having a high ELOC. This was especially true of both mothers and fathers born after 1944 (Table 3). These associations were independent of one another (Table 6), and were not explained by characteristics of the girl’s early childhood (Table 11). Her own year of birth was also a predictor of ELOC − this not only indicates the era in which she was growing up, but also her age at the time her LOC was measured.

There were also associations with young ages of the parents (Table 3). Although at first sight young ages may be thought synonymous with year of birth, in actual fact there is a less than perfect correlation between the two measures (r = −0.82). This can be explained thus: consider the year of birth of a parent *y_p_* and the year of observation *y_ob_* which is fixed for each woman; then the number of years between the two dates equals the age of the parent when the girl was born *a_p_* plus the age of the daughter when her LOC was measured *a_loc_*. Thus:

(*y_ob_* − *y_p_*) = (*a_p_* + *a_loc_*).

Consequently for any parental year of birth, there would be a number of different combinations of ages at which the daughter was born together with the age at which her LOC was measured. For parents born after 1944 they would be young at the birth of their daughter, but so would a proportion of parents born in earlier years.

For this reason the variables (year of birth and age) were treated separately, but the logistic regression analyses demonstrated that parental age showed no independent association once other factors had been taken into account, and therefore that years of birth had important associations.

### 4.2 Parental education

As described above, education level of each parent was measured in regard to educational achievements, characterised as either O-level (or equivalent) and higher, or lower (including no qualifications). The daughters of parents with the higher education levels were each significantly less likely to have an ELOC (Table 3, Table 11) with similar adjusted odds ratios for each parent: mother (1.43 [95% CI 1.27, 1.61]); father (1.37 [95% CI 1.22, 1.54]).

### 4.3 Ethnic background

There were relatively few ethnic minority families residing in the Avon area in the early 1990s, and the few (∼4%) in the current study were from a variety of minority groups including African-Caribbean, Pakistani, Bangladeshi, Indian, Chinese, and African. Division by these groups resulted in numbers that were too small for valid analysis. However when they were grouped together as ‘non-white’, there were no differences in the ELOC proportion when either she, her mother or her father were considered.

### 4.4 Social group

Social group of each parent was based on their last occupation. For women this was problematic as it was the norm for many women to stop work as soon as they were married, and they consequently were not given a classification. We tried to counter this by giving the women an extra category to cover this, but that resulted in the initial positive trend with ELOC (Table 3) failing to enter the early analyses (Table 4). For the fathers, the nature of occupations changed over time so that the results were difficult to interpret. In addition there was a considerable amount of missing information—consequently this variable was omitted from the final model (Table 11) with the consequence that the numbers in the model increased from 6615 to 9286.

### 4.5 Smoking

The smoking habits of the parents were ascertained from the daughters—unadjusted analyses showed that smoking of both mothers and fathers were associated with ELOC (Table 3). In addition, information was obtained concerning whether the mother had smoked when pregnant with her daughter. These were analysed together, with the interpretation that if the mother and/or father smoked this would indicate that the study woman was exposed to environmental tobacco smoke in childhood, whereas if the prenatal smoking was the more relevant variable, then an intrauterine effect might be more relevant. In the event, prenatal smoking was the key variable for the mother (OR 1.23 [95% CI 1.12, 1.35]), but paternal smoking also entered the final model (OR 1.27 [95% CI 1.14, 1.41]).

### 4.6 The woman in infancy

Unfortunately accurate measures of birthweight and gestation were not available for these women. However some details concerning her birth were available. One of the unexpected findings concerned the presence of a birthmark in the first months of life (Table 7); this was robust to adjustment for other features of early childhood (Table 8) but the association was attenuated when characteristics of the parents were taken into account (Table 9, Table 10). Similarly although there was an apparently protective association with breast feeding, it did not survive inclusion of parental smoking habits; this was not surprising since mothers who smoke during pregnancy have been shown to be less likely to breast feed in many population studies (Scott and Binns, 1998).

### 4.7 Home life

Women who were themselves born in the Avon area were much more likely to have an ELOC than were women born elsewhere in England, or even elsewhere in the world (Table 7). This finding was robust to control for other features of early childhood or the characteristics of her parents (AOR 1.56 [95% CI 1.43, 1.71]; Table 11). It suggests that women who migrated to Avon prior to the point at which they completed their LOC score were considerably more internally oriented than their peers who had stayed within the same area throughout.

Other features of early childhood that were linked to increased rate of ELOC included the number of older siblings, especially if more than two, whether her mother had died, whether her parents had divorced or separated during this time period, whether there was a step-father, step-siblings, or her mother’s partner present, and whether she felt that her early childhood was unhappy. Conversely if her parents were present in the home, she was much less likely to have an ELOC (Table 7). On adjustment for the other features of early childhood, the presence of the father and the number of siblings were the variables that predominated in this group (Table 8). Further adjustment for characteristics of the mother and the father (Table 11) indicated that both these factors were robust, with the relationship with older siblings (AOR 1.28 [95% CI 1.15, 1.42]) and absence of her father (AOR 1.81 [95% CI 1.49, 2.19]) being highly significant.

### 4.8 Strengths and limitations

The strengths of this project lie in: (a) the large sample size; (b) the detailed information collected concerning the background of the parents and early childhood of the study subject; (c) the fact that, having developed the model of early factors that are of major importance in regard to the development of an ELOC in adulthood, the next phase of the project will be to determine the features of later childhood and adolescence that may be important, including traumatic events during the period up to 16 years of age. This later analysis will be designed to determine whether the nine factors already identified can be explained as indicators of risk of later events and influences.

There are a number of possible limitations to this study. First, although our analyses allowed for many possible confounders, there may well be other pertinent early exposures that were not considered here. Second, the analyses were restricted to the ∼80% of the pregnant population that took part in the study. We know that those who did not take part were biased in that they were more likely to be teenagers and/or of low educational achievements (features known to be outcomes of a more externalised LOC). Nevertheless the differences in these demographics were relatively small (Fraser et al., 2013). Third, although it is normal to take account of the individual’s current social circumstances and education levels when analysing psychological features of their backgrounds, we deliberately have not done so. We consider this to be very appropriate for looking at the consequences of LOC, but when looking at antecedents, current educational attainments and occupation levels are more likely to be a consequence of LOC orientation—and therefore controlling for such aspects of the individual would have the effect of diminishing any true findings in regard to childhood antecedents. Fourth we have no measures of the LOC of the women’s own parents, which may indicate explanations for some of the findings of this study. Finally the information on the women’s childhood is obtained retrospectively, and may be subject to recall bias. This will be tested in future studies where the antecedents of the offspring of these women will be compared with the information collected prospectively throughout their childhoods.

## 5 Conclusions

Locus of control of reinforcement has been defined as the perception of a connection between one’s actions and their consequences (Rotter, 1966). Measures of internality and externality have been shown to be associated with a number of different factors, including academic achievement, psychological well-being and beliefs (e.g. see Lefcourt, 1983; Nowicki and Duke, 2016). Here we have demonstrated a number of independent influences, the explanation of which requires elucidation. This includes the increase in the women’s ELOC rate with her parents’ year of birth; and the increased rate of ELOC if the woman was born in Avon and remained there until her pregnancy.

## Declarations

### Author contribution statement

Jean Golding: Conceived and designed the experiments; Analyzed and interpreted the data; Wrote the paper.

Steven Gregory: Performed the experiments; Analyzed and interpreted the data; Wrote the paper.

Yasmin Iles-Caven: Wrote the paper.

Stephen Nowicki: Conceived and designed the experiments; Wrote the paper.

### Competing interest statement

The authors declare no conflict of interest.

### Funding statement

This work was supported by a grant from the John Templeton Foundation (Grant ID. 58223). The UK Medical Research Council and the Wellcome Trust (Grant ref: 102215/2/13/2) and the University of Bristol currently provide core support for ALSPAC. The funders had no involvement in the study design nor in the collection, analysis and interpretation of the data.

### Additional information

(TBC) Data associated with this study is available from the ALSPAC Data Archive (http://www.bristol.ac.uk/alspac/researchers/access/).
